# Inferring Broad Regulatory Biology from Time Course Data: Have We Reached an Upper Bound under Constraints Typical of In Vivo Studies?

**DOI:** 10.1371/journal.pone.0127364

**Published:** 2015-05-18

**Authors:** Saurabh Vashishtha, Gordon Broderick, Travis J. A. Craddock, Mary Ann Fletcher, Nancy G. Klimas

**Affiliations:** 1 Department of Medicine, University of Alberta, Edmonton, AB, Canada; 2 Institute for Neuro-Immune Medicine, Nova Southeastern University, Fort Lauderdale, Florida, United States of America; 3 Center for Psychological Studies, Nova Southeastern University, Fort Lauderdale, Florida, United States of America; 4 Miami Veterans Affairs Medical Center, Miami, Florida, United States of America; Fondazione Edmund Mach, Research and Innovation Centre, ITALY

## Abstract

There is a growing appreciation for the network biology that regulates the coordinated expression of molecular and cellular markers however questions persist regarding the identifiability of these networks. Here we explore some of the issues relevant to recovering directed regulatory networks from time course data collected under experimental constraints typical of in vivo studies. NetSim simulations of sparsely connected biological networks were used to evaluate two simple feature selection techniques used in the construction of linear Ordinary Differential Equation (ODE) models, namely truncation of terms versus latent vector projection. Performance was compared with ODE-based Time Series Network Identification (TSNI) integral, and the information-theoretic Time-Delay ARACNE (TD-ARACNE). Projection-based techniques and TSNI integral outperformed truncation-based selection and TD-ARACNE on aggregate networks with edge densities of 10-30%, i.e. transcription factor, protein-protein cliques and immune signaling networks. All were more robust to noise than truncation-based feature selection. Performance was comparable on the in silico 10-node DREAM 3 network, a 5-node Yeast synthetic network designed for **I**n vivo **R**everse-engineering and **M**odeling **A**ssessment (IRMA) and a 9-node human HeLa cell cycle network of similar size and edge density. Performance was more sensitive to the number of time courses than to sample frequency and extrapolated better to larger networks by grouping experiments. In all cases performance declined rapidly in larger networks with lower edge density. Limited recovery and high false positive rates obtained overall bring into question our ability to generate informative time course data rather than the design of any particular reverse engineering algorithm.

## Introduction

Before the emergence of high throughput techniques, biology was deeply entrenched in a reductionist study of one component gene or protein at a time. Though now often depreciated, such studies have provided a wealth of information about the various roles of individual molecular entities. With the advent of high-throughput techniques such as microarray, mass spectrometry, RNA-seq, chip-seq and multi-channel flow cytometry, it is now possible to simultaneously survey many cellular components including mRNA, proteins, and metabolites. There is now a growing appreciation that almost all biological morphologies and functions emerge as a result of complex interactions between constituent molecules or entities [1]. These interactions drive fundamental processes within various intra-cellular compartments, ultimately determining the behavior of a cell as well as the extent and nature of signaling with neighboring cells [2] in both health and disease. As such, understanding these interactions can ultimately lead to more effective clinical treatments. Specifically, an integrative systems approach to biology has the potential to provide new insights into complex illnesses by leveraging broad molecular and cellular surveys [3] to cast various disease-associated genes and related pathways [2] in the proper mechanistic context. However, two major challenges exist: (i) the accurate identification of biological regulatory networks, also called reverse engineering and, (ii) the quantitative study of regulatory network structure and function, as it applies to clinical medicine.

In the past two decades, the reverse engineering of causal gene regulatory networks from time course expression profiles has received special attention with a number of methods and mathematical formulations being proposed for network inference. In S1 Table we present a summary of the principal methods grouped into several broad classes, namely: Logic-based models such as Boolean networks (BN) [4–9], probability based methods such as dynamic Bayesian networks (DBN) [10–17], information theoretic approaches [18, 19], and Ordinary Differential Equations (Linear and Non-linear) based methods [20–28]. In addition, model-free approaches with roots in machine learning/data mining [29–31] and hybrid methods [18, 32–35] have also become popular avenues for the inference of directed biological regulatory networks from time course data. A number of excellent reviews exist that describe the underlying principles, advantages and limitations of various inference methods [36–45]. However, with the exception of the DREAM (Dialogue on Reverse Engineering Assessment and Methods) community-wide challenge [46–50], very few initiatives have sought to compare the relative performance of these methods directly [51–54] and in quantitative terms. They do agree nonetheless that the availability of suitable data constitutes one of the primary obstacles to the more complete and accurate inference of directed biological networks [49]. This is only exacerbated with in vivo human or animal studies where blood can only be sampled at low frequency (10–15 time points) across a relatively short time horizon (24 hours or less). Moreover, for budgetary reasons such detailed studies are typically limited to relatively small subject groups (often less than 20 subjects) examined under a select number of response conditions (often only 1) [55].

It is very important to keep such experimental limitations in mind if in addition to recovering connectivity one is also attempting to infer directed networks for the purpose of designing candidate treatment courses that might be directly predictive of clinical trial outcomes in human subjects. If this is the objective then the method must also allow the user to simulate the dosage and timing of specific network perturbations. While several important inference algorithms have been proposed (S1 Table), many require a quantity and type (e.g. knockout) of data that is more consistent with in vitro experimentation in cell cultures than with in vivo experimentation in human or animal subjects. For example, Boolean network (BN) models allow the user to draw on a broader body of *a priori* knowledge and represent response dynamics as discrete on-off transitions with a simple delay. However BN algorithms such as REVEAL [6] remain computationally expensive and sensitive to experimental noise [56]. Moreover, discrete on-off behavior remains a coarse-grained approximation with a resolution that is typically unsuitable for treatment design beyond the initial exploratory phases. These issues are partially addressed by computationally faster and noise-tolerant probabilistic Boolean networks (PBN) [8, 9] where relationships between variables are captured as joint probability distributions. Dynamic Bayesian networks (DBN) offer further improvements in the analysis of experimental time course data [57] and several algorithms have been proposed including BANJO [10] BNFINDER [14,15], GlobalMIT [13], and DREM [16, 17]. While DBNs can infer feedback loops they continue to require data exceeding that of typical in vivo animal studies despite efforts to control computational complexity with alternative scoring methods such as GlobalMIT [13] and BNFINDER 2 [15]. Similar to DBNs, information theoretic algorithms such as Dynamic CLR [18] and TD-ARACNE [19], have become available to infer regulatory networks from time course data. In general, information-theoretic approaches use a generalization of the pair-wise correlation coefficient called mutual information (MI) [58]. As with the basic Bayesian model, MI is a non-parametric measure, making no assumption regarding the distribution of the data. Although such methods typically require substantial amounts of data, improvements in performance reported for TD-ARACNE are such that we have retained the latter as a candidate method in the current comparative analysis.

Making simplifying assumptions about the distribution of the data allows one to move towards more conventional regression-based models. Perhaps the simplest of these are time-delay forecasting models that are based on Granger causality. With roots in machine learning and data mining these methods are often called model-free since they do not assume any regulatory model structure a priori. However their general applicability is accompanied by a substantial data requirement that matches or even exceeds that of DBNs [59] despite the recent introduction of LASSO penalties [31, 60]. The discrete time equivalent of an ODE, a classical difference equation, can be obtained by restricting this type of forecast model to a single time step lag in the response variable and no lag in the regressor variables. Indeed this is the basic model underlying the TSNI integral method [20] evaluated in this work. Classical continuous ODE models have long been used to describe biochemical reaction kinetics. The data requirements of ODE based methods, at least in their basic linear form, can be quite succinct and this form can readily capture the direction and type of regulation. These models can also be used directly to simulate treatment perturbations making them well suited to support the computational design of clinical interventions. Of the popular ODE-based methods surveyed in S1 Table, some like NIR [22], and the Inferelator [25] require additional prior information, for example the use of gene knockout data, making them less suitable for human *in-vivo* studies. Moving beyond a linear ODE formulation several variants of the nonlinear ODE S-system model have been proposed such as TDSS [27] and NeRDS [28]. However, increases in the number of model parameters leads to a corresponding increase in the data requirements [28]. Moreover this does not necessarily lead to an increase in fidelity as some of the results presented here will show. Hybrid methods combine the strengths of different methodologies. For example, the algorithm proposed in [18] combines the scalability of information theoretic method CLR and causal inference capability of ODE based method, the Inferelator. However, with few exceptions [34], these methods require the expression time course be supplemented with different types of data such as knockout and knockdown data that are not frequently available for human subjects [18, 32, 33].

In the present study, we focus on the inference of local directed regulatory networks from time course data with properties similar to those that might be obtained from animal or human subjects under in vivo conditions. In keeping with this, we have attempted to assess algorithms whose data requirements (data type and quantity) are in line with that typically available from in vivo time course studies and that also support the resolution required to simulate treatment kinetics. Based on our survey of methods, we found ODE-based algorithms like TSNI [24], TSNI integral [20] and the one proposed in Yeung et al. [21], most suitable. The algorithm proposed in [21] and TSNI use similar techniques of Singular Value Decomposition (SVD) and Principal Component Analysis (PCA) for dimensionality reduction. In contrast, TSNI integral uses a forward stepwise regression technique to infer sparse directed networks. Furthermore the conventional gradient formulation of the rate equation is re-written as finite difference equations in TSNI integral to improve performance on noisy experimental data [20]. The architecture of these methods, like most, is based on generic core components that include feature selection and parameter estimation steps. To further explore how performance might be affected by design choices in these component parts we constructed and re-assembled these simple generic building blocks de novo, applying two popular classes of feature selection to a conventional linear ODE model namely the truncation of candidate terms or their projection onto composite constructs. Finally, we also assessed the performance of TD-ARACNE [19] since the latter is reported to have circumvented the typically large data requirements associated with conventional information theoretic methods. Because there are no widely agreed upon benchmark circuits in humans where the true circuit structure is known we used simulated data generated by a gene network simulator NetSim [61] to provide an equitable benchmark. This also made it possible to alter the underlying network size as well as sampling rate and the number of time courses in each data set. To our knowledge this type of standardized comparison focused on methods that are robust to the constraints of in vivo human studies and that also offer sufficient temporal resolution for simulation-based treatment design has not been conducted previously, especially not at the level of the component parts.

We found that all methods performed similarly on noise-free simulated data, with the exception of the information theoretic method TD-ARACNE, which typically exhibited a lower median performance. In selecting ODE model terms, truncation was less tolerant of experimental noise than projection-based approaches. Irrespective of noise levels, we found that all methods were extremely affected by the reduction in network edge density obtained in larger networks. In smaller simulated networks consisting of 5–10 nodes with edge densities similar to typical biological networks (10–30%), values in excess of 0.40 were obtained for the F-score, an aggregate measure summarizing precision and recall. To explore the broader applicability of these results, we assessed the leading ODE-based methods in recovering the DREAM 3 in silico 10-node networks [48], the 5-node Yeast synthetic IRMA network [62] as well as a 9-node human HeLa cell cycle network [30, 63]. Results were comparable to those obtained on NetSim simulated networks of similar size and edge density. All methods were found to be more sensitive to the number of time courses than to sample frequency. Based on the results of this simulation study, at least 10 time course experiments, sampled at 10 time points, would be required to infer a 10 node network with a median recall and median F score of 53%(±3%) and 0.39(±0.04) respectively. In aggregating multiple experiments the most significant improvement in performance was obtained by using the broken stick projection method on groups of 10 or more time course profiles.

It would appear that inference of directed regulatory networks still faces challenges and that less intrusive sampling techniques, i.e. higher frequency, and safer perturbation protocols may be required if we are to infer regulatory networks that fully exploit the breadth of current multiplex surveys. It should be noted that the basic inference models examined in this work are not novel, nor were they intended to be. Instead the analysis conducted here focused specifically on how standard methods might be deployed under conditions typical of in vivo studies in human or animal subjects. Our results emphasize the value of aggregating networks identified from individual time courses and the stratification of subjects into groups. This work also highlighted the importance of tuning the algorithm parameters using a priori simulation of artificial biological networks comparable in size and complexity. Indeed default parameter values do not perform well across a broad range of conditions and even a simple reverse engineering model if tuned correctly has the potential to perform as well as more sophisticated methods. For the moment at least, both the data and the methods appear better suited to the study of individual transcription factor sub-networks, as well as cytokine signaling and flow cytometric studies in groups of experimental subjects.

## Materials and Methods

### Ethical approval

This study received ethics approval by the University of Alberta Health Research Ethics Board (MS4_Pro00018859) and the Miami Veterans Affairs Medical Center Research and Development Committee (file 4987.76)

### Simulated experimental data

To reliably assess the performance of the selected methods we used simulated data such that the true structure of the underlying regulatory network was known, and we could alter network parameters in a controlled manner (network size, time points, noise levels, etc.). All reference networks were created and their behavior simulated using a gene network simulator known as NetSim [61]. NetSim enforces some of the known topological properties of biological regulatory networks such as sparseness, scale-free distribution of connectivity, and clustering granularity independent of the number of nodes. Un-weighted directed networks were produced where the sign of the edge (positive or negative) described the type of regulatory action (+1 for promotion or -1 for inhibition). These network interactions were then translated into fuzzy logic statements by NetSim. The target transition state for a given node at time t+1 is determined by resolving the fuzzy logic statement describing the regulation of that node. A sigmoidal activation function is then used by NetSim to modulate the incremental transition from the node’s current state in the direction of its target state. This incremental change in state is weighted by a time constant capturing both synthesis and degradation dynamics. In all simulations the parameters describing node dynamics were sampled from Gaussian distributions with mean and standard deviation as recommended by the authors. Similarly the initial states were assigned randomly for all nodes at the beginning of each simulation run. Consistent with the current literature (e.g. [19]), we computed all performance metrics based on the direction (source to target) but not the type of interaction (promotion or inhibition). Reverse engineering algorithms are commonly evaluated based on the recovery of regulatory networks using very similar or even identical models as those used in the generation of simulation data. In this work we made concerted efforts to avoid this; using standard ODE and probabilistic models to recover networks from data that was generated by logic-based simulation instead. We consider this to be a more challenging task.

### Selected Network Identification Methods

#### Rate Equation Models

A standard rate equation model is a popular formulation used as the foundation for a broad group of contemporary network identification methods [21–24], including that of Yeung *et al*. [21], Network Identification by multiple Regression (NIR) [22], Mode-of-action by Network Identification (MNI) [64], Time Series Network Identification (TSNI) [24], TSNI integral [20] and others. According to this model, the rate of change in concentration of one gene/transcript/protein can be described through a linear system of ordinary differential equations (ODEs) as a function of the current concentration of other genes/transcripts/proteins as described in Eq 1, where *a*_*i*,j_ is the parameter describing interaction between node *i* and *j*. More precisely, it represents the influence of node *j* on the rate of change of expression of node *i*. A positive value of *a*_*i*,j_ represents activation of node *i* by node *j*, negative value represents inhibition and zero value represents no interaction between node *j* and *i*.

$$\frac{dx_{i}}{dt}=a_{i,1}x_{1}+a_{i,2}x_{2}+⇌\text{K}+a_{i,n}x_{n}$$ (1)

Eq (1) can be rewritten in the matrix form (Eq 2). Here, X is an *n* x *1* vector and A is an *n* x *n* matrix containing the weight of all the edges of the network. This matrix has also been called the adjacency matrix.

$${\dot{X}}^{y}(t)=A\cdot X(t)$$ (2)

Many methods including the popular time-series network identification (TSNI) method proposed in [24] accommodate external perturbations *u(t)* to the system. For example, dose response experiments provide a strong basis for the identification of system dynamics. In accounting for external perturbations, Eq 2 will become,

$${\dot{X}}^{y}(t)=A\cdot X(t)+B\cdot u(t)$$ (3)

Here, *B* is similar in size to *A* and *u(t)* represents the external perturbation at time *t*.

While most ODE-based methods use the instantaneous derivative at time *t*, a recent extension called TSNI integral [20] uses an equivalent model integrated and rewritten as a finite difference equation (Eq 4) as a means of improving robustness in the presence of experimental noise.

$$\overset{0}{\underset{t_{f}}{\int }}{\dot{X}}^{y}(t)\;dt=A\overset{0}{\underset{t_{f}}{\int }}X(t)\;dt\;+\;B\overset{0}{\underset{t_{f}}{\int }}U(t)\;dt,\;f=1,2,\text{K},M$$ (4)

Whether based on the conventional differential equation or the equivalent finite difference equation, the final form is that of a linear regression model where estimates for the values of the unknown parameter sets A and B (Eq 3) are recovered from the experimental data.

In biological systems, the regressor terms in these equations are not expressed independently of one another but rather follow coordinated patterns. Traditional ordinary least squares estimation will generally perform poorly when correlated or collinear terms are used together as these leading to an increase in the uncertainty in parameter estimation referred to as variance inflation [65]. This is typically resolved by one of the two basic approaches namely truncation and projection. The first of these consists in selecting subsets of the original regressors that are minimally redundant. In this work we used a stepwise variable selection [66] method whereby terms were evaluated sequentially based on their respective partial-F test values. Model terms with a p (partial F) < 0.05 were selected for recruitment into the ODE regression model while those currently in the model but showing a revised p (partial F) > 0.10 were pruned.

The second approach consists of projecting the original regressor variables onto a new set of aggregate constructs that are mutually independent. These constructs or latent vectors consist of weighted linear combinations of the original variables and are typically estimated using a diagonal covariance matrix estimate produced by singular value decomposition (SVD) or principal component analysis (PCA) [67]. The most significant of these latent vectors (LV) then serve as a basis for least square regression and the identification of the parameter set A. While there exist as many LV as original variables, the bulk of the shared signal is typically recovered in the first few features. In this work we used an extension of standard PCA called partial least squares (PLS) regression [68, 69] to identify the structure of the LVs. We then evaluated two methods for selecting the number of these features that should be retained, namely the so-called broken stick method, a variant of Horn’s technique [70] for determining significant decrease in the importance of the leading eigenvalues [71], and the Bartlett’s method [72] which evaluates the trailing eigenvalues for equality. These and other methods are reviewed in Jackson (1991b) [73] and more recently Peres-Neto et al., (2005) [74]. Finally, consistent with the compatible literature, we also applied pre-processing of the data that included log transformation and z-score normalization. We also applied a first order hold interpolation model for inferring values between sample points.

#### Parameter tuning of ODE based models

Both stepwise sequential selection and PLS projection methods were evaluated for identification of the conventional ODE model. The finite difference formulation was evaluated as implemented in the TSNI integral method. The latter uses stepwise feature selection in its estimation of the parameter set A. In addition, for this method we used the final prediction error (TSNIF) with the default parameters values with the exception of the parameter ‘restk’ which we tuned to obtain the maximum F score, a combined measure of positive predictive value (PPV) and recall. The ‘restk’ parameter imposes sparseness to the inferred network and therefore affects the F score directly. For more details on TSNI integral, we refer readers to [20]. TSNI integral is freely available at http://dibernardo.tigem.it/softwares/time-series-network-identification-tsni-integral.

In order to assess permutations of the basic algorithmic components associated with the conventional ODE formulation, we implemented these separately rather than use the specific combinations encoded into existing packages. In these implementations we again tuned the algorithm parameters to each problem scenario in an attempt to provide best achievable F score. For example, in the case of stepwise variable selection the null probability threshold values for inclusion and removal into the model (*p*_*enter*_ and *p*_*removel*_) were tuned. To mimic this in the case of projection methods the variable influence on projection (VIP) [68] was used. Based on this metric PLS regression terms were ranked according to the weight of their contribution to the latent vectors capturing the most overall variability in the data. Typically a VIP>1.0 is considered significant; this threshold was optimized here for each scenario. Finally, in order to retain only the most important edges the resulting networks were pruned on the basis of the quantile rank of the edge weight. Here again the quantile threshold applied to the edge weight was tuned for each scenario. This was done using a global optimization method, namely a constrained simulated annealing, to balance computational cost and thoroughness. All algorithms were encoded in MatLab using the functions available in the Statistics Toolbox and the Global Optimization Toolbox (The MathWorks, Inc., Natick, MA).

#### Information Theoretic Time-delay ARACNE (TD-ARACNE)

TD-ARACNE [19] is an extended version of the popular information-theoretic algorithm ARACNe (Algorithm for the Reconstruction of Accurate Cellular Networks) [75] that also retrieves the statistical time dependency between sequential gene expression profiles. Similar to ARACNe, the information theoretic measure of Mutual Information (MI) is used to capture the dependency between two molecular species or network nodes, with statistically independent nodes having a MI value of 0. The MI value between two nodes *i* and *j*, can be represented as described in Eq 5.

$$MI_{ij}=H_{i}+H_{j}-H_{ij}$$ (5)

where H_*i*_ and H_*j*_ are the entropies of nodes *i* and *j*, respectively. Entropy H is defined as follows where *p(x*_*i*_ *)* is the probability that node *i* will assume state *i = 1*:*n*:

$$H(X)=\sum_{i=1}^{n}p(x_{i})\;\log(p(x_{i}))$$ (6)

TD-ARACNE infers directed networks in three steps: 1) detection of the time point corresponding to the initial change in expression for all individual nodes e.g genes, 2) network construction based on pair-wise MI and 3) network pruning. The first step is meant to identify possible regulator nodes or genes based on the sequence of activation. The initial change of expression (IcE) in a sequence of expression values for gene *g*_*a*_ *; g*_*a*_^*0*^, *g*_*a*_^*1*^, *… g*_*a*_^*t*^ can be defined as follows (Eq 7) where *τ*_*up*_ and *τ*_*down*_ are two fold-change thresholds defining relative increase or decrease in expression.

IcE(ga)=argjmin[ga0gaj≥τup or gajga0≤τdown] (7)

In the second step, TD-ARACNE uses bootstrapping to identify significant statistical dependencies between the activation of gene *a* at time *t* and gene *b* at time *t+Δt*. This is subject to the constraint of temporal precedence whereby gene ‘*a’* may only influence gene ‘*b’* if IcE(g_a_) ≤ IcE(g_b_). In the last step TD-ARACNE uses an additional information theoretic measure Data Processing Inequality (DPI) [76] to identify and remove indirect associations, first among synchronously expressed nodes and in a second step across time points. TD-ARACNE is freely available as part of the Bioconductor package and can be downloaded from http://www.bioconductor.org/packages/2.12/bioc/html/TDARACNE.html or from http://bioinformatics.biogem.it. For further details about TD-ARACNE, we refer readers to Zoppoli et al. [19]. In our assessment, all user-adjustable parameters were set to the optimal values recommended by the authors for a network of equivalent node degree.

### Assessing Network Recovery

We assessed the performance of each selected method on the different sizes of networks, noise levels and time points. We use standard statistical measures such as positive predictive value (PPV), recall and F1 score to report the performance for each method. PPV describes the number of correctly identified connections as a fraction of all connections inferred, both correctly (true positive, TP) and incorrectly identified (false positive, FP) (Eq 8). Recall is calculated as the number of connections that were correctly recovered by the algorithm expressed as a fraction of all connections present in the true simulated network, namely those that were recovered (true positive, TP) as well as those that were missed (false negative, FN) (Eq 9).

$$PPV=\frac{\text{Truly inferred connections}}{\text{Total inferred connections}}=\frac{TP}{TP+FP}$$ (8)

$$\text{Recall}=\frac{\text{Truly inferred connections}}{\text{Total no. of connections in true network}}=\frac{TP}{TP+FN}$$ (9)

F1 score (F) is an aggregate measure combining PPV and recall. It is akin to the geometric mean of PPV and recall and can be represented as follows:

$$F=\frac{2(PPV\times \;recall)}{PPV+\;recall}$$ (10)

## Results

### Personalized Networks

Personalized medicine is directed at the identification of illness and intervention at the level of a specific individual. While this is an attractive goal, several questions arise. For example, if we wanted to recover a network from a single experiment for a given individual, how well would we do on average in a diverse population? Moreover, how would the result change for the same person from one day to the next?

#### Mapping individuals with a single time course experiment

To explore the effects of person-to-person variability on network recovery we used NetSim to generate 20 sparse modular networks randomly, composed of 10 nodes each and with similar topological characteristics. Each simulated 10-node network consisted of 10–20 interactions that translated into median edge density of 15.6%. Edge density for a directed network of E edges and N nodes can be calculated as *E/ N(N-1)*. This number of nodes and edge density is consistent with the scale found in synthetic biological networks that have been studied *in vitro*. For example, *E*.*coli* SOS response network that is often used in the evaluation of reverse engineering methods consists 8–9 nodes [19, 24] and the edge density of cortico-cortical fiber tract of the mammalian brain ranges between 10–30% [77]. We used each simulated network to generate a single time course that was sampled at 10, 25 and 50 time points. Expression profiles were generated both with and without 20% Gaussian noise allowing us to assess the effect of sampling frequency and experimental noise respectively on the performance of selected methods (S1 Fig). For noise-free data all methods performed over a narrow range of median F scores (0.2–0.26) (S2 Table). Nonetheless results of a two-way analysis of variance (ANOVA) presented in Table 1 show that F score is significantly affected by the choice of method irrespective of noise (p ≤ 0.001). Though sampling frequency did not affect F scores significantly in the presence of 20% Gaussian noise (p = 0.69) over this initial range of values, this factor did trend towards significance in the absence of noise (p = 0.07). Indeed while the majority of methods appear relatively robust (S1 Fig and S2 Table) the inclusion of noise produced a noticeable decrease in network recall and corresponding significant decrease in the median F score in the case of the stepwise fit method.

**Table 1:** Impact of sample size, experimental noise and algorithm selection on network recovery.

Effect	Sum Sq.	d.f.	Median Sq.	F	Null p
*0% Noise*					
Time points	0.0224	2	0.0112	2.66	0.07
Method	0.0772	4	0.0193	4.57	*0*.*0014*
Method x Time points	0.0313	8	0.0039	0.93	0.49
*20% Noise*					
Time points	0.0046	2	0.0023	0.377	0.69
Method	0.1986	4	0.0497	8.055	*0*.*00*
Method x Time points	0.0494	8	0.0062	1.001	0.44

Results of a two-way ANOVA for the F score obtained by applying the 5 reverse engineering methods to single time course simulations of 20 different sparse and modular 10-node biological networks sampled at 10, 25 and 50 time points, both with and without 20% experimental noise (S1 Fig)

Both Bartlett’s and broken stick methods for the selection of latent features in the ODE projection model produced comparable results in terms of PPV, recall and F score values. On average, both of these methods could infer 70–95% of the true network (median recall). However, this required that 5–7 connections be inferred for every true connection recovered, leading to low median PPV (S2 Table). Slightly better PPV values were obtained with the stepwise fit to the conventional ODE however in the presence of noise this method could only infer 15–30% of the true network leading to lower F scores. The finite equation based TSNI integral provided equivalent or slightly better median PPV but with a loss of approximately 5–30% in coverage of the true network (median recall) resulting median F scores comparable to those obtained with the projection techniques. The information theoretic method TD-ARACNE produced the sparsest estimates of all selected methods. TD-ARACNE was able to infer one true connection out of every 4–6 inferred connections i.e. 18–25% PPV. However, this improvement in PPV cost most of the reference network unrecovered (S2 Table). Also, only minimal effects of noise were observed on PPV, recall and F score at any of the three sampling frequencies for this method.

#### Variations in time course from the same individual

In addition to person-to-person variability we may also expect a slightly different response from the same person on any given day. To explore the consistency of recovery for a specific network from any single experiment we randomly generated a reference network of 10 nodes consisting of 19 interactions (i.e. 21% edge density) with modular topology using NetSim. We then used this fixed reference network to simulate 20 different expression time courses each initiated at random conditions. These were once again sampled at 10, 25 or 50 time points respectively in the presence and absence of 20% noise. Similar trends in performance were observed when using the different methods for the recovery of a single reference network as was observed across the different networks (data not shown). Improved F scores were observed at higher edge density but these remained below 0.40 even in the absence of noise.

#### Scaling to larger networks

Most of the proposed network inference methods have been found to work better on small sub-networks of 5–10 nodes whether they be rate equation-based such as TSNI integral or information theoretic like TD-ARACNE [19, 20]. However, Vinh et al. (2012) [78] questioned how representative the recovery statistics might be on such small networks. To explore this further, we used the above-mentioned methods to recover networks including those composed of a larger number of nodes than that found in the synthetic networks typically reported. We constructed random reference networks composed of 5,10,15, 20, 30 and 50 nodes and with decreasing order of edge densities of 40, 21, 11, 8, 5 and 3% respectively, all with the same properties of sparseness and modularity. Each network was used to generate 20 simulated time course experiments, sampled at 50 time points, where 20% Gaussian noise was added to mimic experimental noise (S2 Fig and S3 Table).

Overall a significant drop in median F-score was observed with increasing number of nodes or decreasing edge density for all the methods (Table 2, p<0.01). Rate equation models, both ODE from projection-based regression and difference equation formulations (i.e. TSNI integral) performed better than TD-ARACNE and step-wise truncation on sparse networks constructed with up to 15 nodes i.e. with 11–40% edge densities. However for networks with edge densities of less than 10% or more than 15 nodes, no noticeable difference in performance was observed among methods (S2 Fig). All methods delivered an F score below 0.10 in their recovery of a 50-node sparse modular network with 3% edge density. In the case of rate equation models identified with projection methods (i.e. ODE-Bartlett and ODE-broken stick) and TSNI integral, the loss in performance was driven mainly by a loss in PPV. This was not the case for the remaining methods, stepwise regression and TD-ARACNe, where both PPV and recall were adversely affected by increasing node degree and decreasing edge density (S3 Table).

**Table 2:** Impact of network edge density and algorithm selection on network recovery.

Effect	Sum Sq.	d.f.	Median Sq.	F	Null p
Method	0.662	4	0.165	61.14	*0*.*000*
Node degree/Edge density	9.312	5	1.862	688.44	*0*.*000*
Method x Node degree	0.598	20	0.029	11.06	*0*.*000*

Two-way ANOVA of F score values corresponding to the recovery of random reference biological networks composed of 5,10,15, 20, 30 and 50 nodes with 40, 21,11, 8, 5 and 3% edge densities respectively, all with the similar properties. Each network was used to generate 20 simulated time course experiments, sampled at 50 time points, where 20% Gaussian noise was added mimic experimental noise (S2 Fig, S2 Table)

### Using repeated time course experiments

As experimental subjects are typically stratified into more homogenous phenotypic groups for study, it may be more relevant to the current realm of clinical research to examine the effects of noise when aggregating several time course experiments in some way. Instead of considering the median performance in recovering the same underlying network at the level of individual time courses we next considered how combining these separate experiments might allow us to make a stronger statement about the group. This could be compared to the difference between narrow patient sub-typing and personalized medicine.

#### Combining networks from the same individual

First, we explored how repeating a challenge multiple times on the same subject might bring us closer to our eventual goal of delivering personalized medicine. We constructed a 10-node reference network with 19 interactions i.e. with 21% edge density and used it to generate 20 time course experiments, each sampled with 50 time points. Next, we added 20% Gaussian noise similar to the previous cases discussed above. We then applied a simple voting scheme to aggregate the networks inferred from each individual time course. A quorum rule was applied to each element across the 20-adjacency arrays. A specific element was conserved in the final consensus array if it was identified as significant in certain minimum number of time course experiments. For example if an edge was present more frequently than a certain threshold, say 12 times across the 20 adjacency matrices, then that edge would be considered as present in the underlying network shared by the grouped experiments. The threshold frequency of occurrence for the selection of edges was determined by optimization on the basis of maximum F score achievable. Typically, a threshold of greater than 50% occurrence was sufficient to deliver stable solutions in inferring a given edge. Because of its higher sensitivity to noise we omitted the stepwise truncation-based approach from this analysis. TD-ARACNE was included despite achieving a poor median recall on single time course experiments to provide a basis for comparison with an information theoretic approach.

In order to examine the effect of group size we repeated our analysis with each selected method on groups of 5, 10, 15 and 20 time course experiments respectively, each sampled at 50 time points (S4 Table). As might be expected the performance of these methods improved when inferring the networks from a group of time courses. Though TSNI integral (S4 Table) was the least responsive when increasing the number of time courses from 1 to 20 trajectories, projection-based approaches overall produced noticeably better F-scores than did TD-ARACNE. With the latter the optimal threshold values for inclusion were so low that the aggregated consensus network approximated the set union of all individually inferred networks. In other words an edge was accepted into the aggregate network if it was present in any one of individual networks. Interestingly, TD-ARACNE continued to show an increasing trend in F-score while the other methods achieved a peak performance at group sizes of 10 or 20 trajectories.

**Fig 1:**
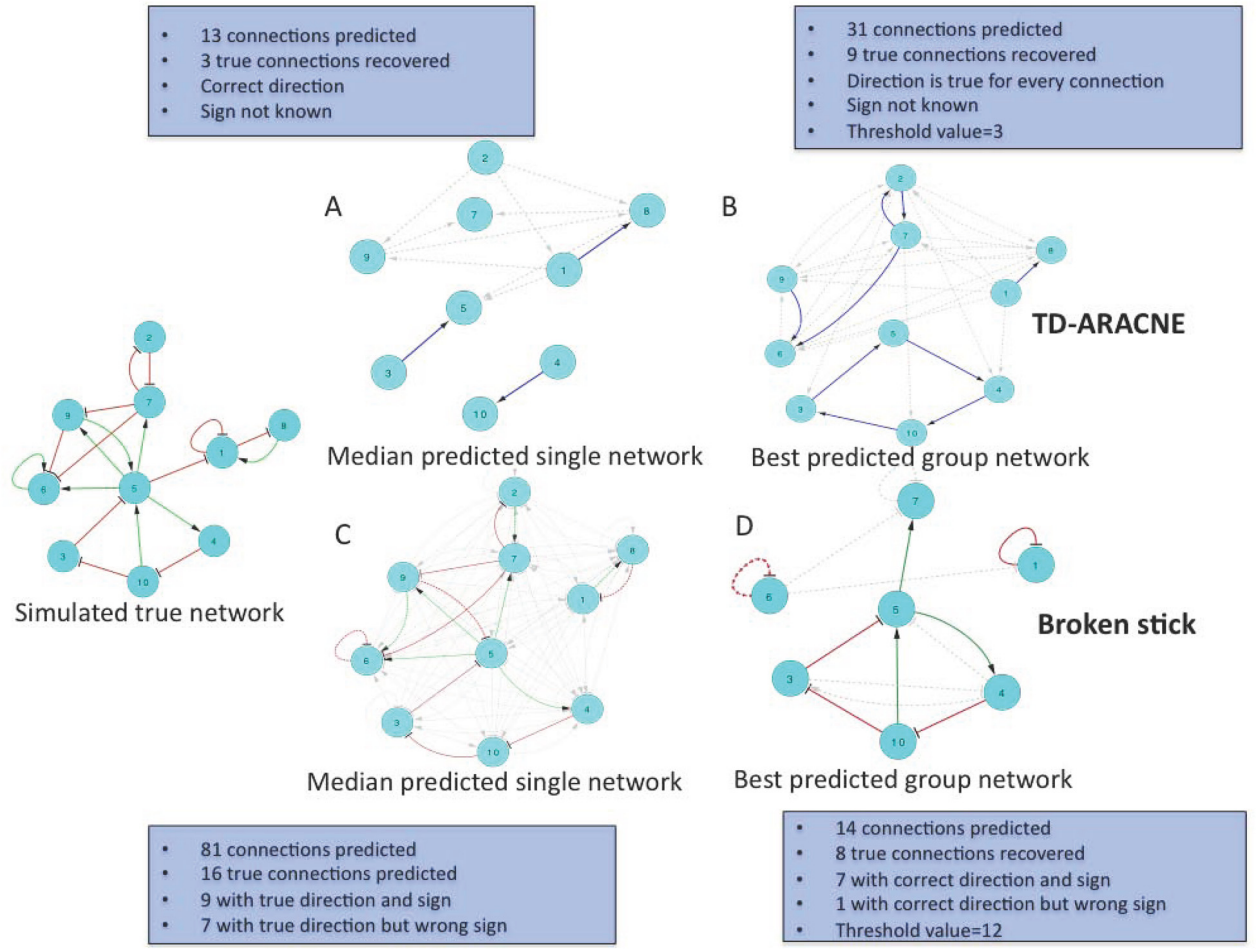
Recovery of an example 10-node network. (A) Median inference by TD-ARACNE using single time course, (B) best possible inference by TD-ARACNE using a group of 15 simulated time courses for the same network, (C) Median inference by broken stick using single time course and (D) best possible inference by broken stick using a group of 15 simulated time courses for the same network.

F-score is a composite measure and the improvement brought about by aggregation can be better visualized using an example network. In Fig 1 we present a simulated 10-node network with 19 connections (21% edge density). A group of 15 time course experiments with 50 time points each were simulated using NetSim. Fig 1A and 1C show one of the 15 inferred networks resembling the median performance of TD-ARACNE and broken stick respectively whereas, Fig 1B and 1D show the consensus networks obtained after the aggregation of 15 inferred networks. In the case of TD-ARACNE, aggregation of networks increases the number of predicted edges whereas this same operation reduced the number of edges predicted in consensus by the broken stick method. At the level of the individual network in Fig 1A, TD-ARACNE predicted very sparse network of 12 connections and predicted 3 out of 19 true connections resulting in median PPV, recall and F score values of 25%, 16% and 0.19 respectively. This performance improved on aggregation of 15 inferred networks where 9 of the 19 true connections were inferred correctly; increasing recall from 16% to 47% (Fig 1B). PPV also increased from 25 to 29%, yielding an aggregate network F score of 0.32. However, the threshold occurrence for the inclusion of an edge in the consensus network was very low i.e. 3 events. In other words, selected edges in the aggregated network were the ones that were inferred in 3 or more networks out of 15 inferred networks. In comparison inference with broken stick achieved median recall of 84% on individual networks i.e. on average 16 out of the 19 true connections could be inferred from a single time course experiment with 50 time points (Fig 1C). Unfortunately in order to predict these 16 true connections (recall = 84%) 65 false positive connections were inferred (grey colored), leading to a PPV of 20%. When aggregating across a group of 15 experiments, the broken stick method inferred a consensus network of 14 connections, 8 of which were present in the true network (Fig 1D). This important reduction in false positive predictions translated into a PPV of 57%. Though some loss in recall (42%) was incurred, the result was nonetheless a net gain in median F score (F = 0.48 vs 0.32). In assessing these performance metrics it is important to remember that we considered only direction of the edges, as is the norm in current literature.

#### Describing illness sub-groups by aggregating networks across individuals

**Fig 2:**
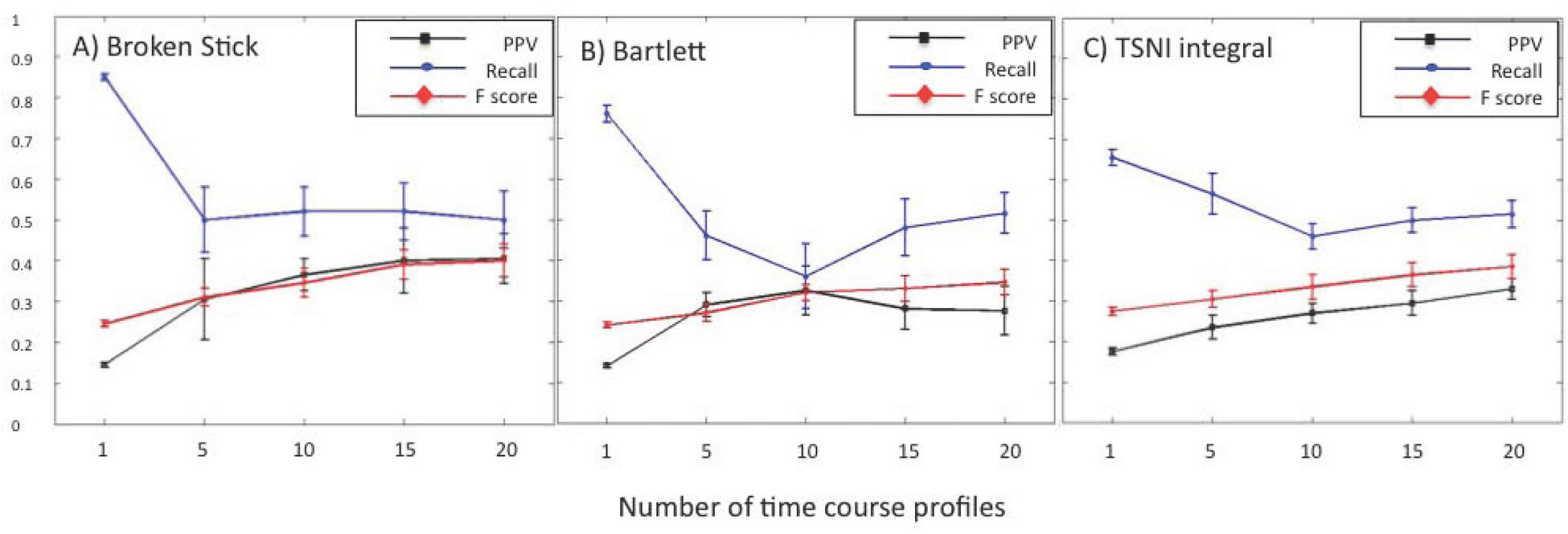
Median performance calculated across subsets from 10 different 10-node simulated networks recovered using (A) Broken stick (B) Bartlett’s and (C) TSNI integral methods applied to groups of time courses . Each network was used to generate groups of 1, 5, 10, 15 and 20 simulated time courses each sampled at 50 time points. All simulations included 20% experimental noise.

To verify how robust these results might be across different networks of the same size we simulated groups of 1, 5,10,15 and 20 time courses, sampled at 50 time points from10 different networks each comprised of 10 nodes with edge densities ranging from 0.10–0.20. These simulated time courses were then analyzed using both projection methods and TSNI integral (Fig 2). Despite being reasonably robust to noise, TD-ARACNE typically lagged projection based methods and TSNI integral in performance (S4 Table and Fig 1). For this reason, we omitted TD-ARACNE from further analysis. Median PPV, recall and F score for each group were calculated along with the standard error. As expected, the greater the number of time courses in a group of diverse individuals, better the performance of the method. However these improvements once again begin to taper off beyond 10–15 time courses. The F score could be improved by a factor of ~1.5 over that of a single time course by using 10 experiments with the broken stick model and 15 experiments for Bartlett’s model and TSNI integral. This improvement in F score for the broken stick method was driven by an initial increase in median PPV at the expense of recall. Similar but less dramatic trends were found for TSNI integral. In contrast, recall values for the Bartlett method recovered and trended positively for group sizes above 10 time courses (Fig 2). Once again while the median performance of TSNI integral was slightly better on single time course experiments, the broken stick projection method delivered equivalent or slightly better performance at all other group sizes (5–20) when sampling 50 time points (Fig 2). A two-way ANOVA of F-score values from grouped data confirmed the significance of this difference in performance between methods (p = 0.008) (Table 3). While the choice of group size also produced a significant affect on F score (p = 0.000), the combined effect of method and group size did not (Table 3). Further analysis revealed that the benefit of increasing group size dissipated quickly and that there was no significant effect (p = 0.40) on F score values for groups of 10 and more time courses. However even at these larger group sizes the choice of method (p = 0.03) continued to be a significant factor (not shown).

**Table 3:** Impact of time course aggregation into subject groups.

Effect	Sum Sq.	d.f.	Median Sq.	F	Null p
Method	0.0726	2	0.0363	4.96	*0*.*0083*
Group size	0.3306	4	0.083	11.3	*0*.*0000*
Method x Group size	0.0245	8	0.003	0.42	0.91

Two way ANOVA of F score values corresponding to the recovery of 10 different networks of 10 nodes with similar properties of sparseness and modularity and with edge densities similar to those of biological networks (10–20%) using groups of 1, 5, 10, 15 and 20 time courses. Each time course was simulated at 50 time points and 20% Gaussian noise was added to mimic experimental noise

#### Sampling for group inference

**Fig 3:**
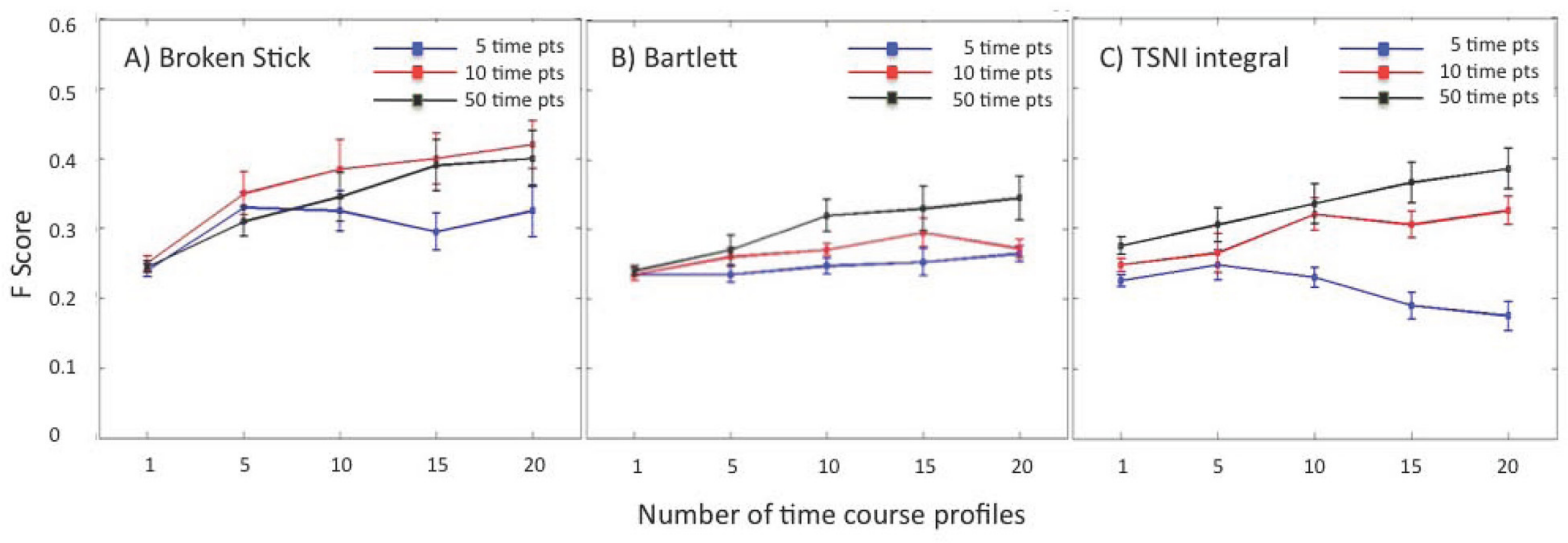
Median F scores calculated on 10 different simulated 10-node networks recovered using (A) Broken stick (B) Bartlett’s and (C) TSNI integral methods on group of expression profiles . Each network was used to generate groups of 1, 5, 10, 15 and 20 simulated time courses each sampled at 5 (blue), 10 (red) and 50 (black) time points. All simulations included 20% experimental noise.

Network recovery in the previous sections was based on the availability of 50 time points. However, in actual in vivo studies the collection of samples at multiple time points is a significant challenge from the perspective of subject well being and cost. To explore the minimum number of time points that might be required by each method, we simulated groups of 1,5,10,15 and 20 time courses sampled at 5, 10 and 50 time points. We found that the broken stick model was less sensitive to the number of time points than the Bartlett and TSNI models. While the former produced the highest F scores when 10 and 50 samples were drawn, this improvement was only noticeable when a group of more than 10 time courses was used (Fig 3). In contrast, drawing 50 samples was uniformly better when using the Bartlett method almost regardless of group size. In the case of TSNI integral, the use of 10 samples was sufficient to produce comparable results, 5 samples being inadequate for all group sizes. These preliminary results suggest that while similarly affected by group size, the broken stick and TSNI integral methods may be fairly tolerant of lower sample frequency. The Bartlett method on the other hand would require the largest number of samples.

### General applicability of the results

#### Generalization of findings to the DREAM challenge

In order to facilitate a broader comparison of these findings we applied this same methodology to the in silico benchmark dataset provided under the DREAM3 sub-challenge for a set of 10-node networks. This dataset was generated using an open source Java tool, GeneNetWeaver [79], and consisted overall of 5 simulated network structures with corresponding steady state and time series data. A modular topology was produced based on patterns of tightly connected gene clusters extracted from experimentally validated regulatory networks. More precisely, 2 out of 5 network structures were based on gene modules extracted from in vivo networks of *E*.*coli* [80] and the other 3 on modules from the *S*.*cerevisiae* in vivo network [12]. A thermodynamic quantitative model of gene regulation that includes both transcription and translation [81] was applied to these network structures to generate dynamic and static simulated experimental data [79].

In keeping with the focus of our current analysis, we used only the dynamic perturbation data. Specifically, we applied both projection-based feature selection techniques along with TSNI integral on the 4 time series provided in DREAM3 for the E.coli2 network of 15 interactions and compared their performance with the median performance obtained using NetSim data for networks with a comparable number of interactions (median interactions = 14). We found that all the selected methods performed similarly in terms of PPV, recall and F score on networks of comparable topology simulated with either NetSim or GeneNetWeaver (S5 Table) suggesting that both simulators are designed to capture the similar network properties.

Further, we compared the performance of these methods with that of Yip et al. (2010) [26], the winning team in the DREAM3 challenge. The latter inferred the underlying networks by combining the inferences obtained from a null hypothesis noise model applied to static knockout/ knock down data, as well as linear and nonlinear ODE models applied to perturbation time course data. Seven different batches were formed consisting of a consensus of different model inferences. We focused first on the results obtained by Yip et al. (2010) [26] using only the time course data to support the consensus of a similar linear ODE model with a more sophisticated nonlinear model (Batch 1 Table 6 of Yip et al., 2010). We then compared the results of assessed ODE models identified from time course perturbation data with that reported in Yip et al. (2010) [26] for the null hypothesis noise model identified from static knockout/ knockdown data (Batch 1 Table 3 of Yip et al., 2010).

When applied to the DREAM3 perturbation time series data, the basic feature selection techniques assessed here based on a simple linear ODE model (broken stick, Bartlett’s and TSNI integral) performed better than the combination of linear and nonlinear ODE models used in Yip et al. (2010) [26] for all networks (Table 4). This being said all ODE models, including those studied in this work, were outpaced by identification based exclusively on homozygous deletion data when inferring sparse networks i.e. networks with 10–15 interactions. However, Bartlett’s and broken stick method of feature selection from time course data approached the performance of the noise model on slightly denser but still sparse networks i.e. Yeast 2 (25 interactions) and Yeast 3 (22 interactions) respectively (Table 4). F scores for the inference of the networks with 10, 15, 22 and 25 interactions were improved with increasing edge density for Bartlett’s (0.22, 0.28, 0.34 and 0.42) and broken stick method (0.19, 0.27, 0.3 and 0.38) respectively whereas that of the noise model fell (Table 4).

**Table 4:** Recovering a 10-node network from a DREAM-3 data set.

E.coli1 (11 interactions)					
Method	Predicted	Correct	PPV	Recall	F score
Yip et. al. Noise model	11	7	0.64	0.64	0.64
Yip et al. linear/nonlinear model	6	1	0.16	0.09	0.12
Broken stick	70	9	0.13	0.82	0.22
Bartlett's method	77	10	0.13	0.91	0.23
TSNI integral	34	4	0.11	0.36	0.17
**E.coli2 (15 interactions)**					
Yip et. al. Noise model	16	12	0.75	0.8	0.77
Yip et al. linear/nonlinear model	5	1	0.2	0.07	0.1
Broken stick	73	12	0.16	0.8	0.27
Bartlett's method	82	14	0.17	0.9	0.28
TSNI integral	31	5	0.16	0.33	0.22
**Yeast 1(10 interactions)**					
Yip et. al. Noise model	11	9	0.82	0.9	0.86
Yip et al. linear/nonlinear model	5	0	0	0	0
Broken stick	72	8	0.11	0.8	0.19
Bartlett's method	83	10	0.12	1	0.22
TSNI integral	26	3	0.11	0.25	0.15
**Yeast 2(25 interactions)**					
Yip et. al. Noise model	13	9	0.69	0.36	0.47
Yip et al. linear/nonlinear model	5	1	0.2	0.04	0.07
Broken stick	71	19	0.26	0.74	0.38
Bartlett's method	83	23	0.28	0.9	0.42
TSNI integral	29	9	0.33	0.36	0.34
**Yeast 3(22 interactions)**					
Yip et. al. Noise model	12	8	0.67	0.36	0.47
Yip et al. linear/nonlinear model	5	4	0.8	0.18	0.29
Broken stick	70	14	0.2	0.61	0.3
Bartlett's method	80	18	0.22	0.8	0.34
TSNI integral	31	7	0.23	0.32	0.27

Comparison of the performance obtained in inferring a 10-node network using the generic methods presented here versus the best performing methods in the DREAM 3 sub-challenge, namely the basic noise model and the combined linear/ nonlinear model.

Marbach et al. 2010 [49] further confirmed that the homozygous deletion data was the most informative of all types of data used in DREAM 3 challenge. However, knockdown/knockout data is not easily accessible in human subjects. In further agreement to the findings of Yip et al. 2010 [26] and Marbach et al. 2010 [49], we also found that complex nonlinear models and/or complex adjustments to linear models did not add significant value to the inference of regulatory interactions and that much simpler more computationally efficient models performed as well when feature selection parameters were optimized based on a priori simulations.

### Recovery of yeast synthetic gene network

As an additional verification of the applicability of the simulated networks used here, we again applied our simple model to data obtained from a synthetic biological network. Cantone et al. (2009) [62] constructed a synthetic network of five genes in the simple eukaryotic organism *Saccharomyces cerevisiae* for **I**n vivo **R**everse-engineering and **M**odeling **A**ssessment (IRMA). This synthetic network includes a variety of regulatory interactions, thus capturing the behavior of larger eukaryotic gene networks on a smaller scale. The network was also designed to be negligibly affected by endogenous genes, and to respond to galactose, which activates transcription of its genes.

We used the switch ‘on’ and switch ‘off’ time series data generated for the IRMA network and inferred the underlying regulatory interactions using projection-based feature selection and TSNI integral. Since the underlying structure of the IRMA network was known a priori we tuned the parameters accordingly for the algorithms in question to optimize network identification based on data averaged across of 5 time courses. All three methods performed comparably in terms of F score on both datasets, with TSNI integral and the Bartlett’s method being the best performers on the switch ‘on’ and switch ‘off’ time series data respectively (Table 5). With the exception of TSNI integral on switch off data, all methods were found to achieve better PPV than a random reconstruction on this synthetic network. Although ODE-based TSNI, the predecessor of TSNI integral was reported by Cantone et al. (2009) [62] to achieve better performance on switch ‘on’ time series (F score = 0.80). However Bartlett’s method (0.67) outperformed TSNI (0.60) on switch ‘off’ time series [62]. Note that the performance of these methods in recovering the 5-node IRMA network appears better than that obtained in the case of 5-node NetSim networks with similar edge density. This is due to different optimization objectives. In the case of the NetSim networks the tuning parameters were optimized to produce maximal median F score on a group of 20 time course profiles whereas the parameter values used in recovering the IRMA network were identified by maximizing F score on a single target time course profile.

**Table 5:** Reconstruction of 5-node synthetic Yeast IRMA network.

	Switch on data			Switch off data		
Method	PPV	Recall	F score	PPV	Recall	F score
Broken stick	0.40(0.5)	0.67(0.67)	0.50(0.57)	0.67(0.67)	0.33(0.33)	0.44(0.44)
Bartlett	0.60(0.75)	0.50(0.5)	0.60(0.55)	0.56(0.56)	0.83(0.83)	0.67(0.67)
TSNI integral	0.40(0.63)	0.83(0.83)	0.53(0.71)	0.29(0.44)	0.67(0.67)	0.40(0.53)
TSNI in Cantone et al.	- (1.00)	- (0.67)	- (0.80)	- (0.75)	- (0.50)	- (0.60)

Broken stick, Bartlett’s and TSNI integral were evaluated on the dynamic data of Yeast 5 node synthetic IRMA network against TSNI performance reported in Cantone et al. 2009 [62]. Values in parentheses show the performance when self-regulation is not considered in the inference as in DREAM 3 challenge.

### Recovery of human gene regulatory network in HeLa cell culture

In addition to the DREAM3 and yeast IRMA data, we also used microarray time course data sampled to characterize periodically expressed transcripts during cell division human HeLa cell line cultures [82]. This data is available at http://genome-www.stanford.edu/Human-CellCycle/HeLa. Sambo et al. 2008 extracted the expression of 9 genes with known and documented interactions in the BIOGRID database (www.thebiogrid.org) sampled at 47 time points from this dataset in their assessment of the reverse engineering method *CNET* [63]. This same time course dataset has since been used for the assessment of several inference methods [30, 31]. It is important to note that the BIOGRID database is updated as new biological knowledge of these interactions becomes available. For example, the network obtained from BIOGRID in [30] is an updated version from the one used in [63] incorporating new interactions. We assessed projection-based feature selection techniques and TSNI integral on the BIOGRID networks used in [63] and [30] respectively. Although this data provides an opportunity to further assess our simulation results in reconstructing a human gene regulatory network it is important to remember that this remains data sampled from an in vitro cell culture system and hence may be sampled at a much higher rate typically available in vivo from human subjects.

**Fig 4:**
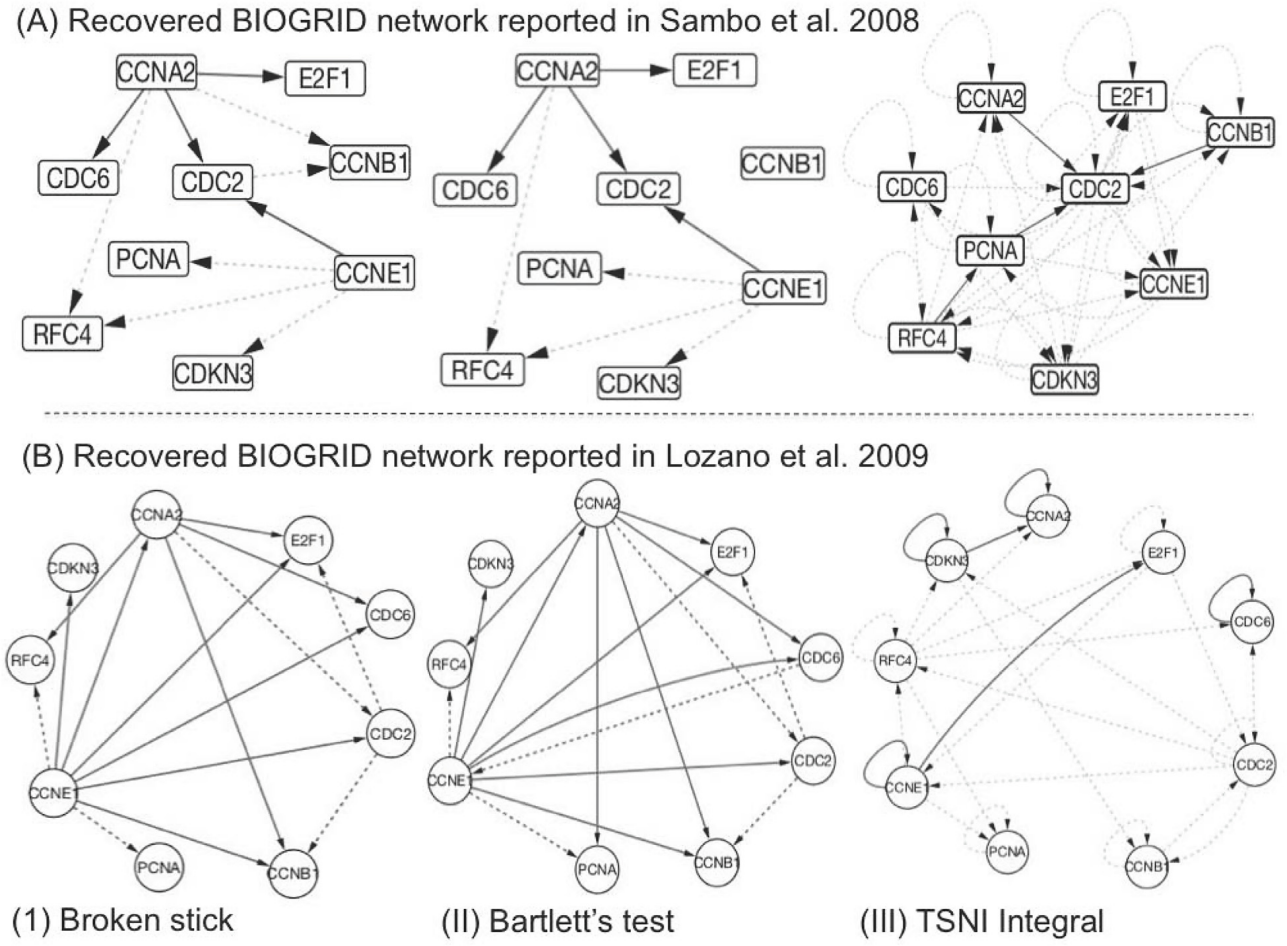
Reconstruction of human HeLa cell cycle network. Directed graphs recovered using (i) Broken stick (ii) Bartlett’s feature selection and (iii) TSNI integral methods applied to the BIOGRID reference network reported in Sambo et al. 2008 and Lozano et al. 2009 (A and B). Solid lines represent correctly inferred interactions (true positives) where as dash lines represent incorrectly inferred connections (False positives).

In our assessment, both projection-based techniques namely, broken stick (F scores = 0.40 and 0.56) and Bartlett’s method (0.47 and 0.60) not only outperform TSNI integral (0.17 and 0.26) in the reconstruction of 9-gene HeLa cell cycle network but also match the performance of methods used in [63] and [30] respectively (Fig 4; Table 6). These results re-affirm that even simple models, if tuned a priori with simulated data, have the potential to perform as well as more complex methods.

**Table 6:** Reconstruction of 9-gene BIOGRID network related to Human HeLa cell cycle.

Network	PPV	Recall	F score
***BioGrid network in Sambo et al*. *2008***			
***C-NET (Sambo et al*. *2008)***	0.36	0.44	0.40
Broken stick method	0.36	0.44	0.40
Bartlett's method	0.50	0.44	0.47
TSNI Integral	0.10	0.44	0.17
***BioGrid network in Lozano et al*. *2009***			
***GGGM (Lozano et al*. *2009)***	0.50	0.72	0.59
Broken stick method	0.63	0.50	0.56
Bartlett's method	0.65	0.55	0.60
TSNI Integral	0.22	0.30	0.26

Recovery of 9-gene BIOGRID network involved in human HeLa cell cycle by applying broken stick and Bartlett’s feature selection methods compared to TSNI integral. These two versions of BIOGRID network were used to assess proposed methods in Sambo et al. 2008 and Lozano et al. 2009 respectively. Reported performance of these methods is also included.

## Discussion

The main purpose of this study was to examine the core design features of some of the basic classes of methods currently available for the reverse engineering of biological networks. In particular, we attempted to gauge their applicability to data collected under experimental constraints typical of in vivo studies where the range of allowable perturbations (e.g. virtual absence of knockout data), the sample frequency and the number of subjects are all significantly limited. Using the two alternative types of parameter estimation commonly applied in the identification of ODE models we assessed the recovery of known networks from simulated perturbation time course data produced by the NetSim platform. Importantly, this was done under a range of sampling frequencies and group sample sizes, key parameters in the design of such experiments. The conventional gradient-based ODE form was also compared to the equivalent time-lagged difference equation (TSNI integral). In addition, the performance of TD-ARACNE, a recently reported information theoretic method adapted for use with time course experiments was also assessed. Finally, the general applicability of our simulation results was explored by reconstructing *in silico* networks using data from the DREAM3 challenge, from the synthetic IRMA network as well as from 9-gene HeLa cell cycle network. In our analysis, none of the methods evaluated performed to the standards of their reported average performance on single simulated time courses created using the logic-based NetSim. It is important to note that in many cases the edge count of the simulated networks was not reported. In addition many of these methods were typically assessed under the more ideal condition where simulated time course data was generated using models similar to those encoded in the reverse engineering algorithm. For example, in evaluating the probabilistic method TD-ARACNe Zoppoli et al. (2010) used a random network to define a set of statistical dependencies then translated these into stochastic differential equations to simulate the actual time course data. Likewise the authors of TSNI integral used very similar differential equation models to generate both the test data and to perform the reverse engineering (Bansal and DiBernardo 2007). Though this is an important departure from real-world conditions it nonetheless offers a possible upper bound for the performance achievable under near ideal conditions. Despite such favorable conditions these reverse engineering methods barely achieve an F score of 0.4 in recovering a 10-node network from 50 time points sampled with 10% noise (based on Table 1 in Bansal and Di Beranardo 2007 and Table 2 in Zoppoli et al. 2010). Based on this body of literature an F score of 0.40 might be considered a near-ideal performance for these methods in recovering networks with small to moderate node degree using single time course data alone.

Here we purposely generated data using a simulation method based on a fuzzy logic framework that differed significantly from the ODE model structure used for reverse engineering. Though still artificial, we consider this situation more realistic. Understandably under these conditions the recovery performance based on single time courses consisted in a median F score of 0.30 or less. Rather than focus on the recovery of networks in individual subjects, we used the commonly accepted practice in human studies of stratifying the cohorts into groups of subjects. In our analysis, projection methods applied to the standard ODE as well as the difference equation model (TSNI integral) were successful in recovering typical biological networks having edge densities of 10–30%, producing a median F score of 0.40 or more when used on groups of time courses. This translated into a predictive precision (PPV) in the range of ~30–40% with recall values between ~50–60% for simulated data from sparsely connected artificial networks designed to exhibit key topological properties similar to those expected in real biological networks. Interestingly this is consistent with values obtained from in vivo regulatory sub-networks surveyed in human immune cells. In recent work, Wang et al. (2009) [83] applied MINDy, an extension of the information theoretic method ARACNe, to the genome-wide identification of modulators of the MYC transcription factor using 254 gene expression sets previously generated for several studies of normal and tumor-related B-cell phenotypes. A literature-based assessment using the Ingenuity software (Ingenuity Systems) revealed that of the 83 reported direct and indirect modulators of MYC expressed in B cells and present on the array, 29 had been recovered correctly, a recall of 35%. Furthermore, 17 of the 35 transcription factors inferred as MYC modulators were either literature-validated (6 or 17%) or had enriched binding sites, leading to an overall upper bound on precision of 48%. It is important to remember that in contrast to these local regulatory modules we intentionally focused in this work on the recovery of networks exhibiting the same edge densities as those observed in broader biological networks, namely 10–30% [77]. We considered this a more challenging task than the recovery of more densely connected sub-networks that are typically used for benchmarking. Indeed, our results show that edge density and performance of the selected network recovery methods are directly proportional with higher values of edge density leading to better performance. The recovery of sparse networks is also highly relevant. Several noteworthy biological networks are known to have low edge density, for example this can be as low as 3.85% between neurons in *C*. *elegans*, and global protein-protein interaction networks in human show edge densities of ~0.4%. Our results suggest that in such sparse networks one might expect reasonable recovery only in the better-connected component sub-networks where edge density is maintained above 10%. Examples of such networks include cytokine signaling between immune cells (60% edge density), cytokine signaling with tissue (40%) and neural networks of cat brain (30% edge density) [84].

For the sparser networks studied here, we found that ODE-based methods generally performed better than TD-ARACNE under conditions that approximated in vivo time-course studies, namely when data collection was restricted to smaller subject groups and infrequent sampling. Among the ODE based methods, stepwise feature selection was less tolerant of experimental noise. Projection-based methods typically faired better as they aggregate terms into composite features, creating an averaging effect that attenuates noise. Unfortunately as we report in this work, projection methods tend to produce far less parsimonious models and a high rate of false positive calls. This is only compounded further in real biological networks where indirect associations may be introduced by unobserved moderators [85, 86]. Inferring networks from a set of expression time course profiles improved the performance of all selected methods. Accordingly, the recovery of personalized networks would require the use of multiple time course experiments applied to the same subject. This is a less than desirable protocol for several reasons. A much more attainable goal consists of grouping patients suffering from the same variant and/or stage of a disease and that share other clinical parameters, such as age, BMI, sex, ethnicity etc. Our analysis suggests that at least 10 time courses would be required for the inference of a representative network for a group of such individuals. Sample frequency is another important design consideration. We found the more parsimonious of the projection approaches i.e. broken stick and TSNI performed consistently when at least 10 time points were used. Our results also suggested that inclusion of additional time course experiments could not correct for insufficient longitudinal sampling.

Nor was there much to be gained on this type of perturbation data by increasing the complexity of the model. Marbach et al., 2010 [49] conducted a comparative analysis of results from all DREAM3 participating teams, some of which used sophisticated non-linear and computationally intensive methods. Though accuracies in excess of 0.60 were produced on small networks (10 nodes) they found that these results were heavily dependent on the type of data available. Indeed, the top five teams all integrated steady-state knockout and knock down data with time-series perturbation data, leveraging the complimentary nature of these data sources. In fact even though both linear and non-linear differential equations were used by the winning team of Yip et al. (2010) [26], the best performance was based on a simple statistical noise model applied solely to steady-state homozygous knockout data. The use of more complex nonlinear ODE forms contributed little if anything towards improving the identification of the underlying networks [49]. Indeed, in single networks of 10 nodes we obtained comparable or better results using a linear ODE model alone on time course perturbation data. Though clearly a very important contributor to the accurate recovery of biological networks, the availability of large-scale deletion libraries in higher mammalian species is currently limited though ongoing efforts focused on the mouse genome are making inroads [87]. Even if such libraries were available for *Homo sapiens*, their analysis supports the broad identification of direct regulatory structure but does not support the identification of co-regulatory motifs nor does it support the identification of regulatory kinetics supporting pharmacokinetic studies. The latter will require kinetic experiments over a range of frequencies as well as dose response methodology [88, 89].

Though preliminary and rooted in a set of basic assumptions regarding the properties of the data, these findings offer an approximate set of guidelines for the design of pilot studies directed at the inference of in vivo network regulatory kinetics as well as some approximate bounds on what we may realistically expect from simple in vivo perturbation studies. Our results and those of others suggest that even in the favorable case of more densely connected sub-networks such as those surrounding transcription factors, the reverse engineering algorithms and the current generation of confirmatory assays may have reached an upper bound in terms of their ability to recover the underlying regulatory network from experimental time course data. This points to the larger issue of information content in the data collected, which is limited by the breadth of experimental conditions that can be safely deployed in human subjects. Despite advances in the reverse engineering algorithms, more informative data sets will be required if we are to realize the potential of personalized network medicine. Algorithms continue to be developed that design new incremental sets of experiments in order to iteratively refine the recovery of the network model [90]. However the data requirements and the extent of the perturbations involved make these more suitable to in vitro studies for the moment. The translation of such methods to human studies will rely on the development of less invasive sampling techniques as well as the development of perturbation techniques that interrogate the physiological system at amplitudes comparable to that of background noise i.e. at scales that are biochemically relevant but not physiologically disruptive [91, 92]. These are typically based on methods adapted from the basic control theory of closed loop identification and are still in their infancy in biology and medicine. In the end, the ultimate bottleneck at the present time may well be our limited ability to generate informative data rather than the design of any particular reverse engineering algorithm.

## Supporting Information

S1 Fig Median performance across a range of networks. 20 different networks of 10 nodes each were used to generate a single time course profile sampled at 10, 25 and 50 time points. Box plots on the left show the median and inter-quartile range of F scores for selected methods on the datasets in the absence of noise. Box plots on the right show the range of F scores for each method on the datasets with 20% random noise added.
(TIFF)

S2 Fig Effect of network scale and edge density on the performance of different methods. For each network scale of node degree between 5 and 50 nodes, a single reference network was created. From each network 20 simulated time courses were obtained using different initial conditions and sampled at 50 time points. All time courses included 20% Gaussian noise. F scores were obtained based on the network recovered from each simulated time course and median values plotted against node scale and with respective edge density.
(TIFF)

S1 File Data for NetSim 10-node time course simulations. Simulated time course data from 20 random NetSim generated 10-node networks, each sampled at 10, 25 and 50 time points with 0% and 20% experimental noise as used in Table 1, S1 Fig and S1 Table. In all data arrays rows represent time point observations and columns represent node state variables.
(MAT)

S2 File Data for 20 NetSim simulated time courses initialized at random conditions. Simulated time course data for 5, 10, 15, 20, 30 and 50-node NetSim networks all sampled at 50 time points with 20% experimental noise as used in Table 2, S2 Fig and S2 Table. In all data arrays rows represent time point observations and columns represent node state variables.
(MAT)

S3 File Data used to demonstrate sample rate and group size effects. Simulated data for 10 different NetSim-generated 10-node networks. Each network was used to generate groups of 5, 10, 15 and 20 simulated time courses each sampled at 5, 10 and 50 time points were selected as reported in Fig 2, Table 3 and Fig 3. All simulations included 20% experimental noise. In all data arrays rows represent time point observations and columns represent node state variables.
(MAT)

S1 Table Review of methods. Review of methods for the reverse engineering of directed networks from time course data published over the past 10 years with the inclusion of select older references describing seminal methods that remain popular.
(XLSX)

S2 Table Summary performance statistics on single time course. Median (a) and mean (b) performance of all selected methods in recovering 20 different 10-node simulated networks, each from a single time course sampled at 10, 25 and 50 time points (S1 Fig).
(DOCX)

S3 Table Impact of increasing network scale. Median (a) and mean (b) performance of all selected methods across different expression profiles for random networks of increasing node degree. Each network was used to generate 20 simulated time course experiments, sampled at 50 time points, where 20% Gaussian noise was added mimic experimental noise (S2 Fig).
(DOCX)

S4 Table Impact of grouping single time courses. Improvement in performance of selected methods by inferring a consensus network for a group of time series experimental data. The reference network consists 10 nodes with 19 edges i.e. (21% edge density). Each experimental time series have 50 time points. A consensus threshold to achieve best possible F score value was used to infer consensus network.
(DOCX)

S5 Table Comparing the performance of methods on NetSim and DREAM3 data. Median PPV, recall and F score obtained by applying broken stick, Bartlett’s and TSNI integral methods on comparable networks of DREAM 3 challenge (E.coli2 with 15 interactions) and NetSim (median value of14 interactions). A set of 20 different networks consisting of 12–17 interactions were simulated by NetSim whereas, 4 time series provided in DREAM 3 challenge were used for E.Coli2 network. Values in parentheses show the performance when self-regulation is not considered.
(DOCX)
