# Supplementary figures and images for: Inferring Broad Regulatory Biology from Time Course Data: Have We Reached an Upper Bound under Constraints Typical of In Vivo Studies?
Source: PLoS One. 2015 May 18;10(5):e0127364. doi: 10.1371/journal.pone.0127364 (PMC4435750; doi:10.1371/journal.pone.0127364)

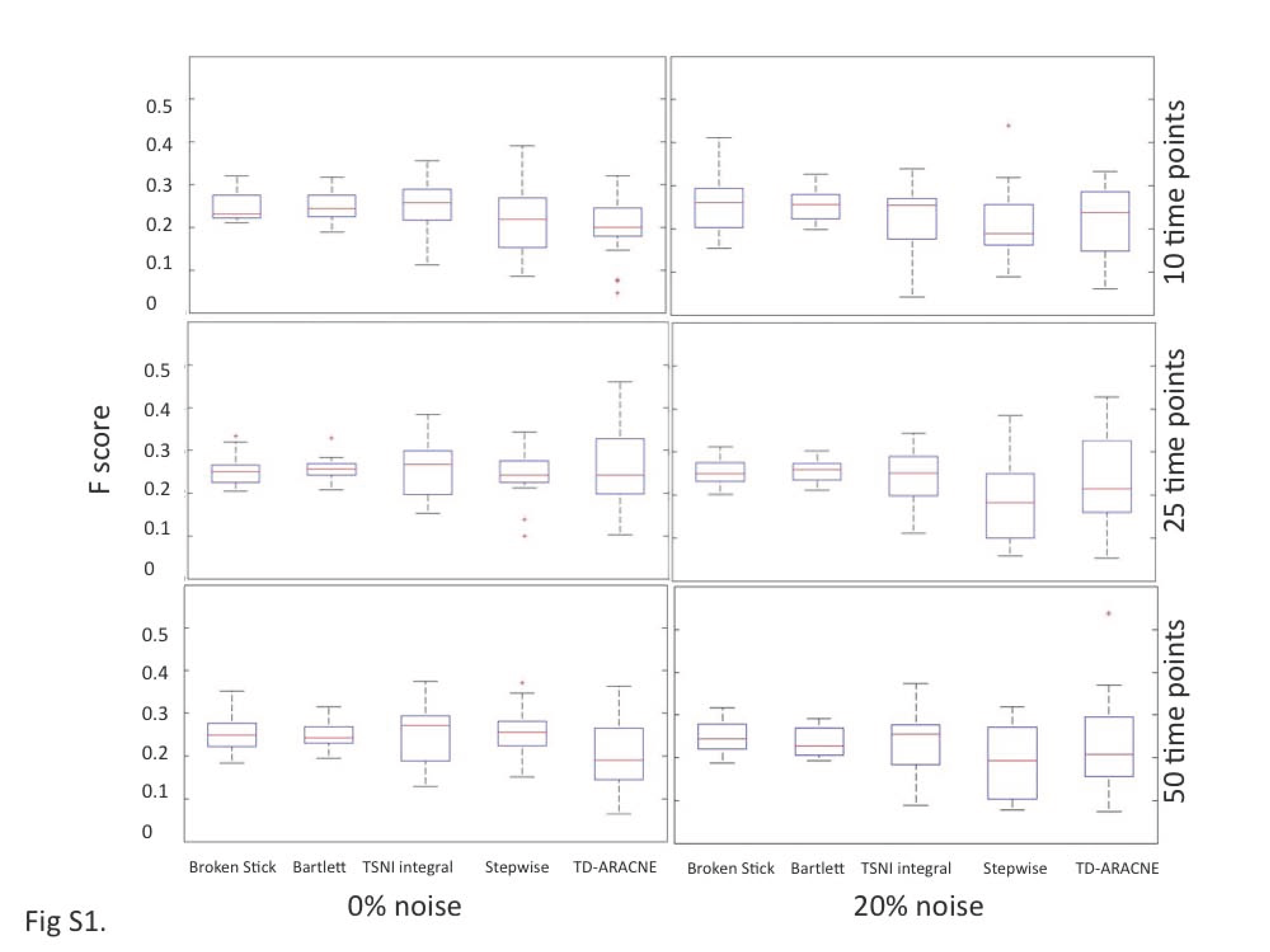

Supplement: S1 Fig — 20 different networks of 10 nodes each were used to generate a single time course profile sampled at 10, 25 and 50 time points. Box plots on the left show the median and inter-quartile range of F scores for selected methods on the datasets in the absence of noise. Box plots on the right show the range of F scores for each method on the datasets with 20% random noise added. (TIFF) [file pone.0127364.s001.tiff]

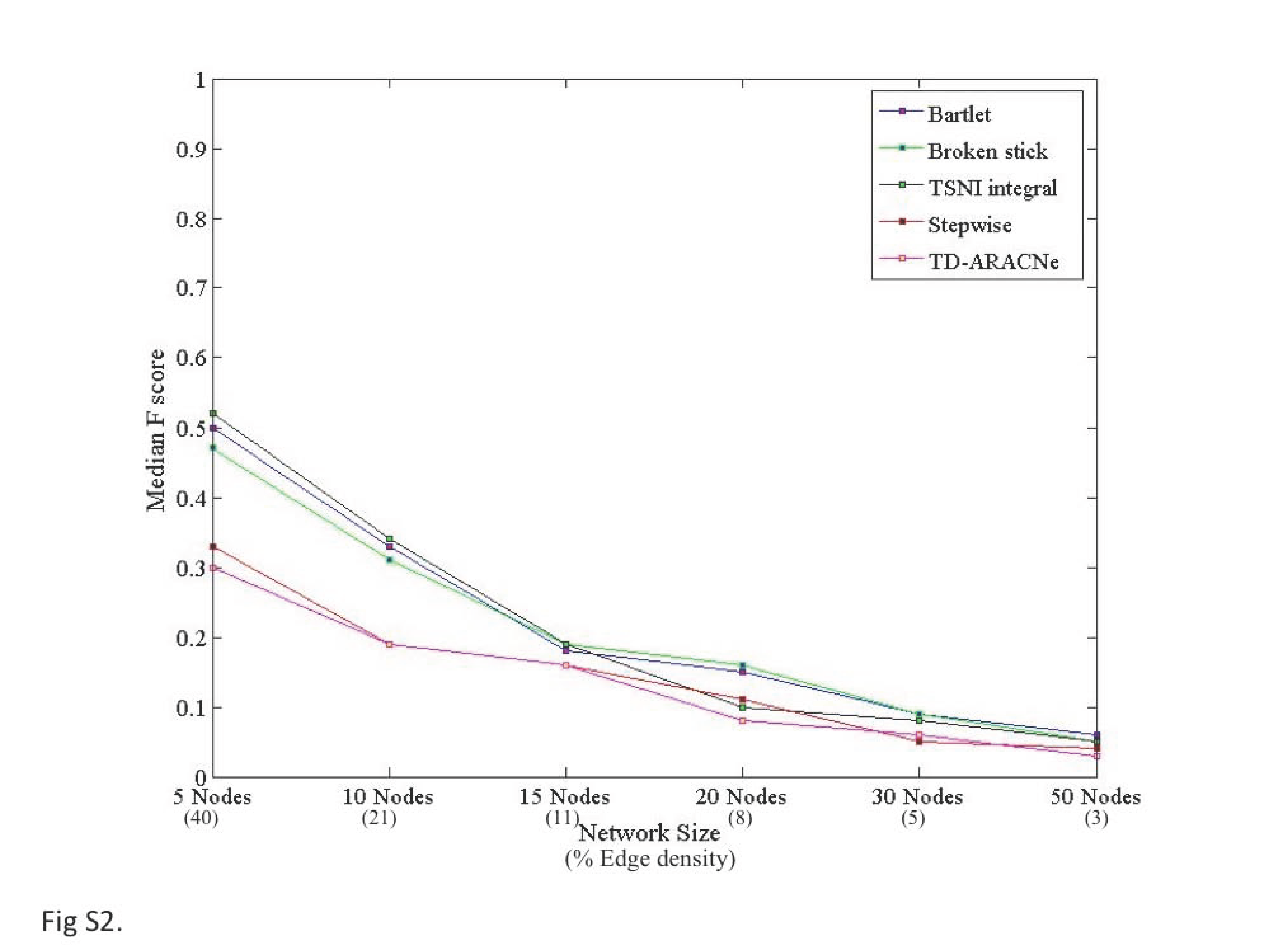

Supplement: S2 Fig — For each network scale of node degree between 5 and 50 nodes, a single reference network was created. From each network 20 simulated time courses were obtained using different initial conditions and sampled at 50 time points. All time courses included 20% Gaussian noise. F scores were obtained based on the network recovered from each simulated time course and median values plotted against node scale and with respective edge density. (TIFF) [file pone.0127364.s002.tiff]
