# Supplementary material for: Inferring Broad Regulatory Biology from Time Course Data: Have We Reached an Upper Bound under Constraints Typical of In Vivo Studies?
Source: PLoS One. 2015 May 18;10(5):e0127364. doi: 10.1371/journal.pone.0127364 (PMC4435750; doi:10.1371/journal.pone.0127364)
Supplement: S2 Table — Median (a) and mean (b) performance of all selected methods in recovering 20 different 10-node simulated networks, each from a single time course sampled at 10, 25 and 50 time points (S1 Fig). (DOCX) [file pone.0127364.s007.docx]

**Table S2a. Summary performance statistics on single time course**

|  | **Methods** | **Time_points** | 10 | | 25 | | 50 | |
| --- | --- | --- | --- | --- | --- | --- | --- | --- |
|  |  | **Noise level (%)** | 0 | 20 | 0 | 20 | 0 | 20 |
| **ODE based method** | **Bartlett's method** | **Median PPV (MAD)** | 0.14 (0.02) | 0.15 (0.02) | 0.15 (0.005) | 0.15 (0.01) | 0.15 (0.01) | 0.14 (0.02) |
|  |  | **Median Recall (MAD)** | 0.85 (0.07) | 0.85 (0.06) | 0.85 (0.07) | 0.93 (0.07) | 0.79 (0.05) | 0.67 (0.09) |
|  |  | **Median F score (MAD)** | 0.24 (0.03) | 0.26 (0.03) | 0.26 (0.01) | 0.26 (0.02) | 0.24 (0.02) | 0.23 (0.03) |
|  | **Broken stick** | **Median PPV (MAD)** | 0.14 (0.01) | 0.16 (0.03) | 0.15 (0.01) | 0.15 (0.01) | 0.15 (0.02) | 0.15 (0.02) |
|  |  | **Median Recall (MAD)** | 0.83 (0.05) | 0.65 (0.09) | 0.73 (0.06) | 0.85 (0.08) | 0.79 (0.08) | 0.79 (0.07) |
|  |  | **Median F score (MAD)** | 0.23 (0.02) | 0.26 (0.04) | 0.25 (0.02) | 0.25 (0.02) | 0.25 (0.03) | 0.24 (0.03) |
|  | **TSNI integral** | **Median PPV (MAD)** | 0.17 (0.02) | 0.17 (0.02) | 0.17 (0.03) | 0.16 (0.03) | 0.18 (0.04) | 0.16 (0.02) |
|  |  | **Median Recall (MAD)** | 0.61 (0.13) | 0.5 (0.09) | 0.65 (0.12) | 0.55 (0.12) | 0.59 (0.12) | 0.59 (0.09) |
|  |  | **Median F score (MAD)** | 0.26 (0.04) | 0.25 (0.04) | 0.27 (0.04) | 0.25 (0.05) | 0.27 (0.06) | 0.26 (0.03) |
|  | **Stepwise** | **Median PPV (MAD)** | 0.18 (0.04) | 0.15 (0.03) | 0.16 (0.02) | 0.21 (0.06) | 0.16 (0.02) | 0.2 (0.09) |
|  |  | **Median Recall (MAD)** | 0.31 (0.11) | 0.27 (0.07) | 0.57 (0.07) | 0.17 (0.09) | 0.64 (0.13) | 0.15 (0.09) |
|  |  | **Median F score (MAD)** | 0.22 (0.06) | 0.19 (0.04) | 0.24 (0.03) | 0.18 (0.08) | 0.26 (0.03) | 0.19 (0.09) |
| **Inform. Theor. method** | **TD-ARACNE** | **Median PPV (MAD)** | 0.17 (0.05) | 0.22 (0.05) | 0.23 (0.12) | 0.23 (0.08) | 0.2 (0.07) | 0.19 (0.07) |
|  |  | **Median Recall (MAD)** | 0.25 (0.07) | 0.24 (0.08) | 0.28 (0.09) | 0.23 (0.09) | 0.2 (0.08) | 0.21 (0.05) |
|  |  | **Median F score (MAD)** | 0.2 (0.03) | 0.24 (0.05) | 0.24 (0.07) | 0.22 (0.07) | 0.19 (0.06) | 0.21 (0.05) |

**Table S2b. Summary performance statistics on single time course**

|  | **Methods** | **Time_points** | 10 | | 25 | | 50 | |
| --- | --- | --- | --- | --- | --- | --- | --- | --- |
|  |  | **Noise level (%)** | 0 | 20 | 0 | 20 | 0 | 20 |
| **ODE based method** | **Bartlett's method** | **Mean PPV (SE)** | 0.15 (0.004) | 0.15 (0.006) | 0.15 (0.004) | 0.15 (0.004) | 0.15 (0.005) | 0.14 (0.005) |
|  |  | **Mean Recall (SE)** | 0.85 (0.02) | 0.85 (0.02) | 0.86 (0.02) | 0.93 (0.02) | 0.81 (0.02) | 0.69 (0.02) |
|  |  | **Mean F score (SE)** | 0.25 (0.007) | 0.26 (0.008) | 0.26 (0.005) | 0.26 (0.006) | 0.25 (0.007) | 0.24 (0.008) |
|  | **Broken stick** | **Mean PPV (SE)** | 0.15 (0.005) | 0.16 (0.01) | 0.15 (0.005) | 0.15 (0.004) | 0.15 (0.006) | 0.15 (0.005) |
|  |  | **Mean Recall (SE)** | 0.82 (0.02) | 0.65 (0.03) | 0.76 (0.02) | 0.82 (0.02) | 0.8 (0.02) | 0.81 (0.02) |
|  |  | **Mean F score (SE)** | 0.25 (0.007) | 0.25 (0.01) | 0.25 (0.008) | 0.25 (0.007) | 0.25 (0.009) | 0.25 (0.008) |
|  | **TSNI integral** | **Mean PPV (SE)** | 0.16 (0.01) | 0.14 (0.01) | 0.16 (0.01) | 0.16 (0.01) | 0.16 (0.01) | 0.15 (0.01) |
|  |  | **Mean Recall (SE)** | 0.61 (0.04) | 0.41 (0.04) | 0.65 (0.03) | 0.53 (0.03) | 0.56 (0.03) | 0.6 (0.04) |
|  |  | **Mean F score (SE)** | 0.25 (0.01) | 0.21 (0.02) | 0.25 (0.01) | 0.24 (0.02) | 0.25 (0.02) | 0.24 (0.01) |
|  | **Stepwise** | **Mean PPV (SE)** | 0.17 (0.02) | 0.17 (0.015) | 0.16 (0.009) | 0.2 (0.03) | 0.16 (0.008) | 0.22 (0.04) |
|  |  | **Mean Recall (SE)** | 0.32 (0.03) | 0.29 (0.03) | 0.56 (0.03) | 0.16 (0.02) | 0.63 (0.04) | 0.15 (0.02) |
|  |  | **Mean F score (SE)** | 0.22 (0.02) | 0.21 (0.02) | 0.25 (0.01) | 0.19 (0.02) | 0.25 (0.01) | 0.19 (0.02) |
| **Inform. Theor. method** | **TD-ARACNE** | **Mean PPV (SE)** | 0.17 (0.02) | 0.19 (0.02) | 0.25 (0.03) | 0.23 (0.03) | 0.21 (0.03) | 0.22 (0.07) |
|  |  | **Mean Recall (SE)** | 0.23 (0.025) | 0.22 (0.03) | 0.26 (0.03) | 0.25 (0.03) | 0.19 (0.02) | 0.22 (0.05) |
|  |  | **Mean F score (SE)** | 0.2 (0.02) | 0.22 (0.02) | 0.26 (0.02) | 0.24 (0.02) | 0.2 (0.02) | 0.23 (0.05) |
