# Supplementary material for: Inferring Broad Regulatory Biology from Time Course Data: Have We Reached an Upper Bound under Constraints Typical of In Vivo Studies?
Source: PLoS One. 2015 May 18;10(5):e0127364. doi: 10.1371/journal.pone.0127364 (PMC4435750; doi:10.1371/journal.pone.0127364)
Supplement: S3 Table — Median (a) and mean (b) performance of all selected methods across different expression profiles for random networks of increasing node degree. Each network was used to generate 20 simulated time course experiments, sampled at 50 time points, where 20% Gaussian noise was added mimic experimental noise (S2 Fig). (DOCX) [file pone.0127364.s008.docx]

**Table S3a. Impact of increasing network scale**

| **Methods** | | **Network size** | 5 Nodes | 10 Nodes | 15 Nodes | 20 Nodes | 30 Nodes | 50 Nodes |
| --- | --- | --- | --- | --- | --- | --- | --- | --- |
|  |  | **Edge density (%)** | 40 | 21 | 11 | 8 | 5 | 3 |
| **ODE based method** | **Bartlett's method** | **Median PPV (MAD)** | 0.35 (0.03) | 0.21 (0.02) | 0.1 (0.002) | 0.08 (0.0009) | <0.1 (0.002) | <0.1 (0.0004) |
|  |  | **Median Recall (MAD)** | 0.87 (0.13) | 0.76 (0.08) | 0.91 (0.04) | 0.9 (0.03) | 0.78 (0.02) | 0.93 (0.014) |
|  |  | **Median F score (MAD)** | 0.5 (0.04) | 0.33 (0.02) | 0.18 (0.004) | 0.15 (0.0015) | <0.1 (0.004) | <0.1 (0.0009) |
|  | **Broken stick** | **Median PPV (MAD)** | 0.32 (0.03) | 0.19 (0.01) | 0.12 (0.01) | <0.1 (0.006) | <0.1 (0.004) | <0.1 (0.003) |
|  |  | **Median Recall (MAD)** | 0.88 (0.13) | 0.84 (0.05) | 0.52 (0.04) | 0.74 (0.05) | 0.82 (0.05) | 0.4 (0.04) |
|  |  | **Median F score (MAD)** | 0.47 (0.04) | 0.31 (0.02) | 0.19 (0.02) | 0.16 (0.01) | <0.1 (0.007) | <0.1 (0.006) |
|  | **TSNI integral** | **Median PPV (MAD)** | 0.4 (0.05) | 0.25 (0.025) | 0.13 (0.02) | <0.1 (0.01) | <0.1 (0.006) | <0.1 (0.004) |
|  |  | **Median Recall (MAD)** | 0.75 (0.13) | 0.53 (0.05) | 0.35 (0.04) | 0.26 (0.05) | 0.33 (0.06) | 0.2 (0.03) |
|  |  | **Median F score (MAD)** | 0.52 (0.07) | 0.34 (0.03) | 0.19 (0.03) | 0.1 (0.02) | <0.1 (0.01) | <0.1 (0.007) |
|  | **Stepwise** | **Median PPV (MAD)** | 0.5 (0.17) | 0.23 (0.05) | 0.19 (0.03) | <0.1 (0.02) | <0.1 (0.01) | <0.1 (0.008) |
|  |  | **Median Recall (MAD)** | 0.25 (0.06) | 0.16 (0.05) | 0.13 (0) | 0.14 (0.03) | <0.1 (0.02) | <0.1 (0.03) |
|  |  | **Median F score (MAD)** | 0.33 (0.05) | 0.19 (0.05) | 0.16 (0.02) | 0.11 (0.02) | <0.1 (0.009) | <0.1 (0.01) |
| **Information theoretic method** | **TD-ARACNE** | **Median PPV (MAD)** | 0.5 (0.17) | 0.25 (0.11) | 0.14 (0.06) | 0.12 (0.06) | <0.1 (0.04) | <0.1 (0.01) |
|  |  | **Median Recall (MAD)** | 0.25 (0.13) | 0.16 (0.08) | 0.17 (0.04) | <0.1 (0.03) | <0.1 (0.02) | <0.1 (0.01) |
|  |  | **Median F score (MAD)** | 0.3 (0.1) | 0.19 (0.1) | 0.16 (0.06) | 0.08 (0.03) | <0.1 (0.02) | <0.1 (0.009) |

**Table S3b. Impact of increasing network scale**

| **Methods** | | **Network size** | 5 Nodes | 10 Nodes | 15 Nodes | 20 Nodes | 30 Nodes | | 50 Nodes | |
| --- | --- | --- | --- | --- | --- | --- | --- | --- | --- | --- |
|  |  | **Edge density (%)** | 40 | 21 | 11 | 8 | 5 | | 3 | |
| **ODE based method** | **Bartlett's method** | **Mean PPV (SE)** | 0.33 (0.01) | 0.2 (0.006) | 0.1 (0.0007) | 0.08 (0.0006) | | <0.1 (0.0008) | | <0.1 (0.0001) |
|  |  | **Mean Recall (SE)** | 0.89 (0.03) | 0.76 (0.02) | 0.93 (0.007) | 0.91 (0.009) | | 0.79 (0.01) | | 0.93 (0.006) |
|  |  | **Mean F score (SE)** | 0.48 (0.01) | 0.32 (0.01) | 0.19 (0.001) | 0.14 (0.001) | | <0.1 (0.002) | | <0.1 (0.0003) |
|  | **Broken stick** | **Mean PPV (SE)** | 0.33 (0.01) | 0.2 (0.004) | 0.11 (0.004) | <0.1 (0.002) | | <0.1 (0.0009) | | <0.1 (0.001) |
|  |  | **Mean Recall (SE)** | 0.86 (0.03) | 0.84 (0.01) | 0.51 (0.02) | 0.75 (0.02) | | 0.82 (0.02) | | 0.4 (0.02) |
|  |  | **Mean F score (SE)** | 0.47 (0.01) | 0.33 (0.006) | 0.19 (0.006) | 0.16 (0.004) | | <0.1 (0.002) | | <0.1 (0.002) |
|  | **TSNI integral** | **Mean PPV (SE)** | 0.38 (0.01) | 0.24 (0.01) | 0.12 (0.008) | <0.1 (0.004) | | <0.1 (0.003) | | <0.1 (0.001) |
|  |  | **Mean Recall (SE)** | 0.73 (0.03) | 0.51 (0.02) | 0.32 (0.02) | 0.28 (0.02) | | 0.33 (0.02) | | 0.2 (0.009) |
|  |  | **Mean F score (SE)** | 0.5 (0.02) | 0.33 (0.01) | 0.18 (0.01) | 0.1 (0.007) | | <0.1 (0.005) | | <0.1 (0.002) |
|  | **Stepwise** | **Mean PPV (SE)** | 0.47 (0.05) | 0.25 (0.02) | 0.18 (0.01) | <0.1 (0.006) | | <0.1 (0.005) | | <0.1 (0.003) |
|  |  | **Mean Recall (SE)** | 0.23 (0.025) | 0.16 (0.01) | 0.13 (0.01) | 0.14 (0.01) | | <0.1 (0.006) | | <0.1 (0.008) |
|  |  | **Mean F score (SE)** | 0.31 (0.03) | 0.19 (0.01) | 0.15 (0.01) | 0.11 (0.008) | | <0.1 (0.004) | | <0.1 (0.004) |
| **Information theoretic method** | **TD-ARACNE** | **Mean PPV (SE)** | 0.61 (0.06) | 0.25 (0.03) | 0.15 (0.02) | 0.13 (0.02) | | <0.1 (0.009) | | <0.1 (0.004) |
|  |  | **Mean Recall (SE)** | 0.21 (0.02) | 0.16 (0.02) | 0.2 (0.02) | <0.1 (0.007) | <0.1 (0.006) | | <0.1 (0.003) | |
|  |  | **Mean F score (SE)** | 0.3 (0.02) | 0.19 (0.02) | 0.17 (0.02) | 0.08 (0.008) | <0.1 (0.005) | | <0.1 (0.002) | |
