# Supplementary material for: Inferring Broad Regulatory Biology from Time Course Data: Have We Reached an Upper Bound under Constraints Typical of In Vivo Studies?
Source: PLoS One. 2015 May 18;10(5):e0127364. doi: 10.1371/journal.pone.0127364 (PMC4435750; doi:10.1371/journal.pone.0127364)
Supplement: S4 Table — Improvement in performance of selected methods by inferring a consensus network for a group of time series experimental data. The reference network consists 10 nodes with 19 edges i.e. (21% edge density). Each experimental time series have 50 time points. A consensus threshold to achieve best possible F score value was used to infer consensus network. (DOCX) [file pone.0127364.s009.docx]

**Table S4**. **Impact of grouping single time courses**.

| Method | Group size | PPV | Recall | F score | %Increase F score |
| --- | --- | --- | --- | --- | --- |
| Bartlett's | 20 | 0.41 | 0.37 | 0.39 | 3-30% increase |
|  | 15 | 0.36 | 0.53 | 0.43 |  |
|  | 10 | 0.24 | 0.63 | 0.35 |  |
|  | 5 | 0.24 | 0.58 | 0.34 |  |
|  | 1* | 0.21 | 0.76 | 0.33 |  |
|  |  |  |  |  |  |
| Broken stick | 20 | 0.47 | 0.42 | 0.44 | 22-41% increase |
|  | 15 | 0.57 | 0.42 | 0.48 |  |
|  | 10 | 0.31 | 0.79 | 0.44 |  |
|  | 5 | 0.24 | 0.84 | 0.38 |  |
|  | 1* | 0.19 | 0.84 | 0.31 |  |
|  | | | | |  |
| TSNI integral | 20 | 0.31 | 0.58 | 0.4 | 12-24% increase |
|  | 15 | 0.3 | 0.68 | 0.41 |  |
|  | 10 | 0.32 | 0.63 | 0.42 |  |
|  | 5 | 0.3 | 0.53 | 0.38 |  |
|  | 1* | 0.25 | 0.53 | 0.34 |  |
|  | | | | |  |
| TD-ARACNE | 20 | 0.31 | 0.47 | 0.38 | 63-100%increase** |
|  | 15 | 0.29 | 0.47 | 0.36 |  |
|  | 10 | 0.21 | 0.58 | 0.31 |  |
|  | 5 | 0.24 | 0.53 | 0.33 |  |
|  | 1* | 0.25 | 0.16 | 0.19 |  |
| * The PPV, recall and F score values reported are equal to the median values | | | | | |
| obtained from the 20 time courses of above mentioned network.  ** Consensus threshold is very low. Almost approximates Union of all networks. | | | | |  |
