# Supplementary material for: Inferring Broad Regulatory Biology from Time Course Data: Have We Reached an Upper Bound under Constraints Typical of In Vivo Studies?
Source: PLoS One. 2015 May 18;10(5):e0127364. doi: 10.1371/journal.pone.0127364 (PMC4435750; doi:10.1371/journal.pone.0127364)
Supplement: S5 Table — Median PPV, recall and F score obtained by applying broken stick, Bartlett’s and TSNI integral methods on comparable networks of DREAM 3 challenge (E.coli2 with 15 interactions) and NetSim (median value of14 interactions). A set of 20 different networks consisting of 12–17 interactions were simulated by NetSim whereas, 4 time series provided in DREAM 3 challenge were used for E.Coli2 network. Values in parentheses show the performance when self-regulation is not considered. (DOCX) [file pone.0127364.s010.docx]

**Table S5. Comparing the performance of methods on NetSim and DREAM3 data**.

|  | Broken stick | | Bartlett's method | | TSNI Integral | |
| --- | --- | --- | --- | --- | --- | --- |
|  | NetSim data | Dream3_data | NetSim data | Dream3_data | NetSim data | Dream3_data |
| Median PPV | 0.15(0.16) | 0.15(0.16) | 0.15(0.16) | 0.15(0.17) | 0.15(0.15) | 0.15(0.21) |
| Median recall | 0.84(0.84) | 0.80(0.80) | 0.93(0.92) | 0.90(0.90) | 0.50(0.50) | 0.40(0.40) |
| Median F score | 0.25(0.26) | 0.25(0.27) | 0.26(0.27) | 0.26(0.28) | 0.23(0.23) | 0.22(0.27) |
